# Prevalence and Incidence of Diabetes in Stockholm County 1990-2010

**DOI:** 10.1371/journal.pone.0104033

**Published:** 2014-08-14

**Authors:** Tomas Andersson, Anders Ahlbom, Cecilia Magnusson, Sofia Carlsson

**Affiliations:** 1 Institute of Environmental Medicine, Karolinska Institutet, Stockholm, Sweden; 2 Center for Occupational and Environmental Medicine, Stockholm County Council, Stockholm, Sweden; 3 Centre for Epidemiology and Community Medicine, Stockholm County Council, Stockholm, Sweden; 4 Department of Public Health Sciences, Karolinska Institutet, Stockholm, Sweden; University of Chieti, Italy

## Abstract

**Background:**

Diabetes is on the rise in the western world, but data from Scandinavia are inconsistent with indications of stable or even reverse trends. To shed new light on this issue, we investigated diabetes trends in Stockholm County 1990–2010, taking into account trends in risk factors and mortality.

**Methods:**

We used data from a large population-based quadrennial public health survey conducted between 1990 and 2010 in Stockholm County (∼2.1 million inhabitants), supplemented with data from national registers. The age-standardized prevalence and incidence rates of diabetes and related risk factors 1990–2010 were estimated in adult inhabitants. We also modelled the influence of potential risk factors on the diabetes trends and estimated the life time risk of diabetes.

**Results:**

The prevalence of diabetes was 4.6% (95% confidence interval (CI); 4.5–4.8%) in 2010 compared to 2.8% (95% CI; 2.3–3.5%) in 1990. Between 1990 and 2002 the prevalence rose annually by 3.8% (95% CI; 2.1–5.5). Incidence rates showed a similar pattern and rose by 3.0% (95% CI; 0.3–5.7%) annually 1990–2002. The rising incidence was mainly attributable to increasing prevalence of overweight/obesity, which rose by 46% during the observation period. In 2010, the lifetime risk of adult onset diabetes was 28% (95% CI; 26–31%) in men and 19% (95% CI; 17–21%) in women.

**Conclusions:**

Diabetes rates have been increasing in Stockholm over the last decades, both in terms of incidence and prevalence, and this development is most likely the result of increasing overweight and obesity in the population.

## Introduction

Diabetes is on the rise world wide; according to the latest report from the International Diabetes Federation, the global prevalence will rise from 8.3% in 2013 to 11.1% in 2033 and the number of people affected by the disease will increase by 57% from 382 to almost 600 million [1]. Less is known about the incidence of diabetes but findings from the US [2] and UK [3] suggest that it is increasing as well, and that the obesity epidemic contributes to this rise [4].

To what extent diabetes is increasing in Scandinavia is unclear; previous studies have yielded inconsistent results with reports of both stable [5]–[7] and increasing [8]–[10] prevalence. Incidence estimates are also conflicting; with indications of constant [5], [10] and even reduced incidence, at least during the last decade [8]–[9]. This is somewhat surprising, since levels of obesity has been rising in Scandinavia [11], although less dramatic than in other parts of Europe and the U.S. Part of these discrepancies may stem from the fact that the risk of diabetes is not uniform in the population but varies considerably by age, sex and lifestyle. The role of such factors for the diabetes trends in Scandinavia is however largely unexplored. Also, since the prevalence depends both on incidence and survival, prevalence of diabetes could rise even if incidence is not, as a result of improved survival in people with diabetes. Swedish data on this topic is however contradictory; one study indicated improved survival primarily in women [12], another study suggested the opposite [13] and yet another study indicated no improvement in survival for people with diabetes over the last decades [14].

Our aim was to study trends in prevalence and incidence of diabetes in Stockholm County 1990–2010, and to estimate driving forces in terms of risk factors and mortality.

## Materials and Methods

### Stockholm Public Health Survey

**Figure 1:**
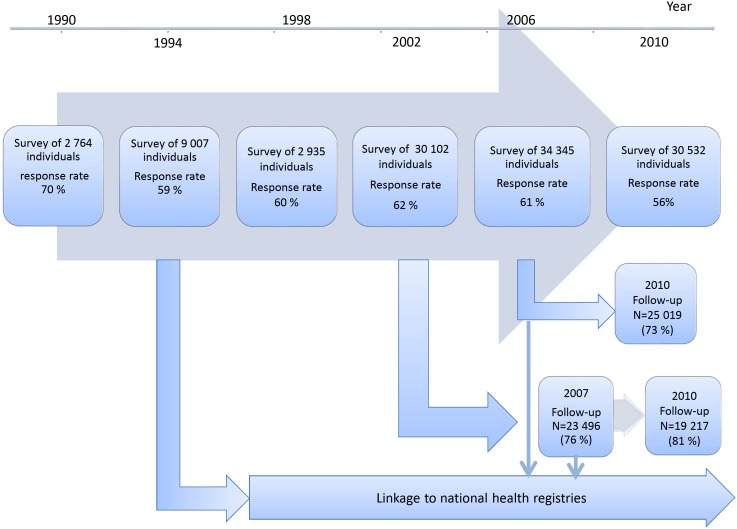
Collection of cross sectional and follow-up data in the Stockholm Public Health Survey.

The health of the adult inhabitants of Stockholm has been investigated in the Stockholm Public Health Survey, every fourth year since 1990 [15]. The survey featured an extensive questionnaire which covers health, demographic, socioeconomic, and lifestyle factors. Participants were selected as a stratified random sample of the inhabitants aged ≥18 years, living in Stockholm County, an urban region including 24 municipalities with approximately 2.1 million inhabitants (∼1/5 of the Swedish population). The sample size has varied from 3930 in 1990 to 55 341 in 2010 with a response rate ranging from 56–70% (figure 1). Investigations of the representativeness of these samples show that responders are more likely to be female, born in Sweden and have higher education and income than the general population [15]–[16]. Eligible for the present investigation were all participants with complete information on diabetes status. The study was approved by the regional ethical board in Stockholm. All data was handled anonymously and no individual could be identified.

### Follow-up

Based on these surveys, three cohorts can be formed and followed prospectively (figure 1); Cohort 1 consists of participants in the 1994 survey who were followed in the national cause- of death registry for the period 1994–2007, using the personal id number assigned to every Swedish Citizen; Cohort 2 consists of participants in the 2002 survey (n = 31 182) who were re-investigated by questionnaire in 2007 (n = 23 794) and again in 2010 (n = 19 327) (figure 1). Correspondingly, participants in the 2006 survey (n = 34 707) were re-examined in 2010 (n = 25 167) (cohort 3). The follow-up of participants in the surveys of 2006 and 2007 also included linkage to the national cause- of death registry (-2011) and prescribed drug registry (2005–2012).

### Diabetes information

Information on diabetes status was assessed by the question “Have you been diagnosed with diabetes?”. For participants in the cohorts, information on diabetes could also be assessed through the national drug prescription registry for the period 2006–2010. This register records all prescription drugs for the entire Swedish population since 1 July 2005 [17]. The drugs are classified according to the Anatomical Therapeutic Chemical (ATC) classification system [18] and those belonging to ATC group 10 (insulin and oral antidiabetic drugs) where used in this study.

### Risk factors

Self-reported information on height and weight was used to calculate body mass index (BMI) as kg/m^2^ which was categorized as normal weight (<25), overweight (25.0–29.9) and obesity (≥30). Information on past and current smoking was used to classify subjects as current, former, or never smokers. Physical activity was assessed through a question on average physical activity during leisure time. There were seven response options ranging from 0 to >5 hours per week. For comparison of the prevalence of physical inactivity between 1990 and 2010, subjects reporting less than 1 hour of physical activity per week were classified as sedentary.

Data on the age and sex distribution of the population in Stockholm between 1990 and 2010, together with the annual number of diseased in each municipality, were obtained from Statistics Sweden (www.scb.se).

### Statistical analyses

#### Prevalence

Using data from the Stockholm Public Health Survey (numbers displayed in table S1), we modelled the age-, sex-, year- and municipality specific prevalence estimates for the years 1990–2010 by logistic regression. These were pooled to yield an overall prevalence estimate for every year between 1990 and 2010 standardised according to the age distribution of the population in Stockholm in 2010 (Statistics Sweden). The regression model contained age, modelled continuously as a third degree polynomial, together with year, municipality and an interaction term for age*year. Prevalence estimates were modelled separately in men and women and with sexes combined. 95% confidence limits were estimated through basic boot-strap with 1000 case resamples [19]. To estimate the linear and segmented trend 1990–2010 we used meta-regression, based on the estimated prevalences and their variances, calculated from the confidence limits [20].

#### Mortality

Data from the three cohorts were used to model the age specific relative risk (RR) of death (all-cause) in people with vs. without diabetes for the period 1994–2012, using a discrete time proportional hazards model allowing for updating of calendar year, age and diabetes status [20]. The model included age (as an ordinal variable), year, diabetes (yes/no) and interaction terms for age*diabetes and year*diabetes. Inclusion of duration of diabetes would have yielded a more precise model, but information on age at onset was not available.

### Incidence

We approximated the annual incidence of diabetes with the use of a) the estimated annual prevalence and b) relative mortality in people with diabetes and c) census data on population size and annual number of deaths, using the formula described in File S1. Confidence limits and trends were estimated using the same method as described above. To estimate the incidence rate attributable to overweight, we extended the model for prevalence with a variable indicating BMI level (<25, 25.0–29.9 and ≥30) and predicted the incidence 1990–2010 under the assumption that no one was overweight. The ratio between the predicted and overall incidence was interpreted as an estimation of the proportion of new cases not attributed to overweight. Cumulative risk of diabetes, defined as the likelihood of developing diabetes between the age 18 and 88, was calculated with the life table method (File S1), using the estimated age-specific incidence of diabetes, and mortality of the Stockholm population of 2010. All analyses were conducted with SAS 9.3 (SAS Institute, Cary, NC, USA).

## Results

### Prevalence of diabetes 2010

In 2010, the overall prevalence of diabetes was estimated at 4.6% (95% CI; 4.5–4.8%) in Stockholm, which corresponds to 73 000 affected inhabitants. The disease was more common in men (5.4%, 95% CI; 5.2–5.6%) than in women (3.9%, 95% CI; 3.8–4.1%) and as expected, the prevalence varied substantially by age and BMI; 14.7% of men and 12.7% of women with obesity (BMI≥30) reported diabetes. Diabetes was also more prevalent in people with low education or low socioeconomic status, in smokers and in those born outside of Sweden (Table 1). In participants born outside of Sweden, prevalence of overweight was 15% higher and prevalence of obesity was 32% higher than in Swedish born participants.

**Table 1:** Estimated prevalence of diabetes by lifestyle and demographic factors. Stockholm Public Health Survey 2010.

	Men		Women	
	N (%)	% Diabetes(95% CI)	N (%)	% Diabetes(95% CI)
**Age**				
18–44	10 076 (51.6%)	1.1 (0.9–1.4)	14 569 (49.2%)	1.2 (1.0–1.4)
45–64	12 382 (32.3%)	7.2 (6.7–7.7)	15 401 (31.4%)	4.5 (4.1–4.9)
65–88	10 048 (16.0%)	15.3 (14.6–16.2)	11 463 (19.3%)	10.1 (9.5–10.7)
**BMI**				
<25	13 867 (48.6%)	2.7 (2.5–3.0)	24 208 (64.5%)	2.0 (1.8–2.1)
25–29.9	13 793 (40.3%)	5.8 (5.4–6.2)	11 198 (25.3%)	5.2 (4.8–5.7)
≥30	3 940 (11.0%)	14.7 (13.6–15.9)	4 602 (10.3%)	12.7 (11.7–13.7)
**Socioeconomic status**				
Manual workers	8 535 (32.9%)	5.5 (5.1–5.9)	9 006 (7.9%)	4.4 (4.0–4.9)
Non-manual employees	17 398 (58.1%)	4.8 (4.4–5.1)	25 774 (68.4%)	3.5 (3.3–3.7)
Professionals, Self-employedand farmers	3 324 (9.7%)	5.6 (4.8–6.5)	1 788 (4.2%)	3.0 (2.2–3.8)
**Education**				
Primary school	5 425 (17.6%)	7.1 (6.5–7.8)	2 625 (16.7%)	6.6 (6.0–7.2)
Upper secondary school	13 357 (40.2%)	6.3 (5.8–6.7)	3 636 (38.3%)	4.5 (4.1–4.8)
University	13 385 (42.8%)	3.7 (3.4–4.0)	9 499 (45.7%)	2.4 (2.2–2.7)
**Country of birth**				
Sweden	27 356 (83.4%)	4.9 (4.6–5.1)	34 398 (82.2%)	3.6 (3.4–3.8)
Europe	3 064 (8.5%)	8.4 (7.4–9.4)	4 595 (10.4%)	5.2 (4.5–5.8)
Outside of Europe	2 086 (8.1%)	7.5 (6.5–8.6)	2 440 (7.3%)	5.9 (4.9–6.8)
**Smoking**				
never	17 423 (60.9%)	3.7 (3.4–4.0)	22 799 (58.4%)	3.6 (3.4–3.9)
former	11 428 (28.7%)	8.4 (7.9–8.9)	13 379 (29.7%)	4.4 (4.0–4.7)
current	3 296 (10.3%)	7.0 (6.2–7.9)	4 796 (11.9%)	4.1 (3.5–4.7)
**Physical activity (hours/week)**				
<1	13 264 (38.5%)	7.3 (6.9–7.8)	16 978 (40.7%)	5.2 (4.9–5.5)
1–2	12 370 (38.5%)	4.5 (4.1–4.9)	17 734 (43.3%)	3.1 (2.9–3.4)
≥3	6 375 (22.9%)	3.5 (3.1–3.9)	5 969 (16.0%)	2.6 (2.2–3.0)

### Prevalence of diabetes 1990–2010

**Figure 2:**
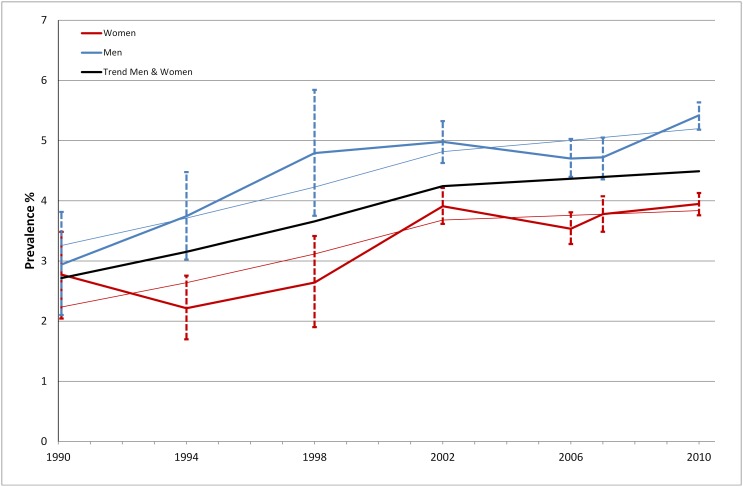
Prevalence of diabetes in men and women 1990–2010.

Prevalence of diabetes was 2.8% (95% CI; 2.3–3.5%) in 1990. Between 1990 and 2002, the prevalence of diabetes rose annually by 3.8% (2.1–5.5%); 3.3% (95% CI; 1.1–5.6% in men and 4.2% (95% CI; 2.0–6.5%) in women (Figure 2). The rise was less pronounced between 2002 and 2010; the annual change in prevalence was estimated at 0.7% (95% CI; −0.7–2.2%). Based on the crude prevalences in 1990 and 2010, i.e. without adjusting for changes in the age structure of the population, and information on population size from statistics Sweden, we estimated that the number of people with diabetes in Stockholm has increased by 103% between 1990 and 2010, which corresponds to approximately 37 000 patients in Stockholm County.

Data from the drug prescription registry, available for the entire population of Stockholm, support these findings and indicate a rise in pharmacologically treated diabetes from 4.4% to 5.0% in men and from 3.2% to 3.5% in women ≥20 years old in Stockholm County between 2006 and 2010. The overall prevalence of drug treated diabetes was 4.2% in 2010 compared to 3.8% in 2006. For the entire Swedish population, corresponding estimates were 4.5% (2006) vs. 5.1% (2010).

### Mortality 1990–2010

The relative excess mortality in people with diabetes declined annually by 1.6% (95% CI; −3.6–1.3%) between 1994 and 2011. In 1990, the relative mortality risk associated with diabetes at age 50 was estimated at 4.0 compared to 2.9 in 2010. Correspondingly, at age 80, the relative mortality risk declined from 2.4 in 1990 to 1.7 in 2010. Improved survival was seen in both men and women, although slightly more pronounced in the latter group (results not shown).

### Incidence of diabetes 1990–2010

**Figure 3:**
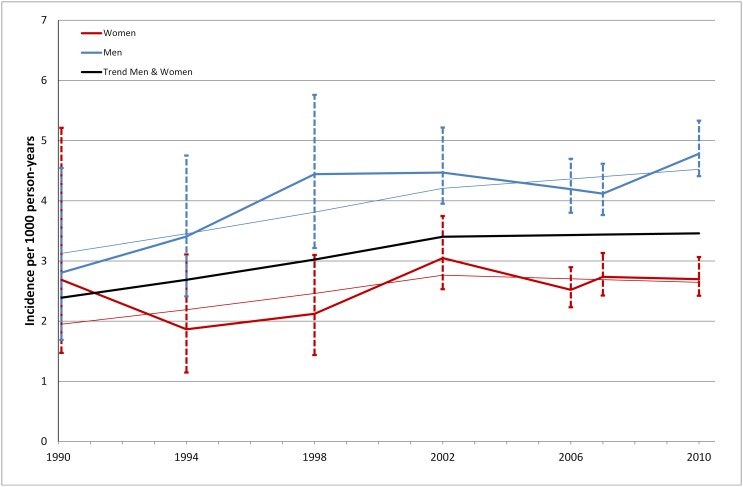
Incidence of diabetes in men and women 1990–2010.

In 2010 the incidence of diabetes in Stockholm was estimated at 3.6 (95% CI; 3.4–3.9) per 1000; 4.8 (95% CI; 4.4–5.3) in men and 2.7 (95% CI; 2.4–3.1) in women (Figure 3). The corresponding rates in 1990 were 2.6 (95% CI; 1.7–3.6) per 1000; 2.7 (95% CI; 1.5–5.2) in women and 2.8 (95% CI; 1.7–4.5) in men. Between 1990 and 2002, the incidence rose at an annual rate of 3.0 (95% CI; 0.3–5.7), but appeared to stabilise from 2002; the annual change in incidence 2002–2010 was estimated at 0.2% (95% CI; −1.5–2.0%). Similar results were seen in men and women. Based on the estimated incidence of diabetes in 2010 and age-specific mortality rates from the population registry in Stockholm for the same year, we estimated that 28% (95% CI; 26–31%) of all men and 19% (95% CI; 17–21) of all women will develop diabetes sometime between age 18 and 88.

### Prevalence of overweight/obesity, physical inactivity and smoking 1990–2010

**Figure 4:**
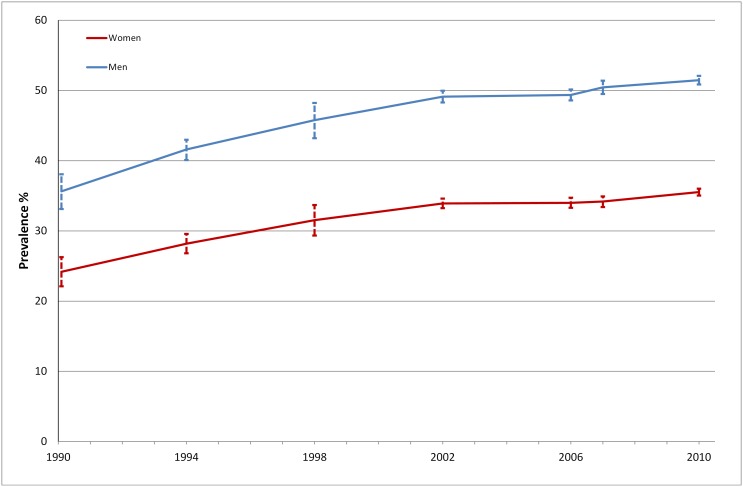
Prevalence of overweight 1990–2010. Stockholm Public Health Survey.

**Figure 5:**
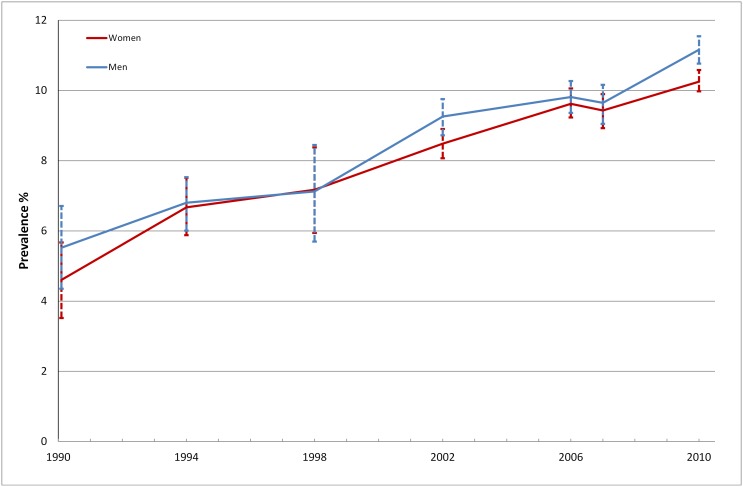
Prevalence of obesity 1990–2010. Stockholm Public Health Survey.

During the period 1990–2010, the prevalence of overweight increased from 35.6% to 51.5% in men and from 24.2 to 35.5% in women (Figure 4). An even more pronounced increase is seen for obesity; from 5.5 to 11.2% (men) and from 4.6 to 10.3% (women) which corresponds to a more than doubling of the prevalence of obesity in the population between 1990 and 2010 (Figure 5). It is noteworthy that more men than women were overweight, whereas the proportion with obesity was similar. The prevalence of obesity in subjects with diabetes was estimated at 31.8% in 2010 compared to 9.0% in 1990. We estimated that of all incident cases in 2010, 41% could be attributed to overweight compared to 18% in 1990. This corresponds to an incidence rate of 1.47 per 1000 (2010) vs. 0.50 per 1000 (1990). Under the assumption that the prevalence of overweight had been stable during the observation period, we estimate that there would have been no rise in the incidence of diabetes.

Between 1990 and 2010, the proportion who reported that they were sedentary almost doubled, from 21.5% to 38.5% in men and from 25.4% to 40.7% in women. Smoking decreased during this period; in 1990, 25.7% of women and 23.6% of men were smokers compared to 10.3% of men and 11.9% of women in 2010.

## Discussion

Our findings indicate that the prevalence of diabetes increased from 2.8 to 4.6% in Stockholm between 1990 and 2010, and the number of people with diabetes doubled. Part of this rise was attributed to increasing incidence which rose by 38% between 1990 and 2002. Overweight and obesity rose in parallel, and our findings indicate that without this negative development, diabetes incidence would have remained stable. Diabetes is a major threat to public health, the estimated risk of developing diabetes between age 18–88, was one in four men and one in five women.

The prevalence of diabetes was 5.4% in men and 3.9% in women in 2010. This confirms that diabetes is more common in men than in women [1] and also that diabetes is less frequent in Scandinavia compared to other parts of Europe [1], [3] and the US [1], [2]. In contrast to some [5]–[7], but not all [9]–[10] previous Swedish studies, our findings indicate that the prevalence rose between 1990 and 2002 at an annual rate of 3.8%. This suggests that Stockholm has had a similar development as other parts of Europe including Denmark [8] and the UK [3]. Contributing to this development was a rise in the incidence of diabetes; between 1990 and 2002 we observed an annual increase of 3.0% in Stockholm. Similar findings were reported from Denmark; Carstensen et al [8] showed that the incidence increased by 5% per year between 1995 and 2004. Increasing incidence has also been reported from other parts of Europe [3]–[4], [22] and the US [2]; In a Scottish study, a doubling of the incidence of type 2 diabetes was seen 1993–2004 [22] and in the UK, incidence of type 2 diabetes rose by two thirds 1996–2005 [3] and in the US incidence of diabetes doubled 1980–2011 [2].

Both in the UK [4] and the US [2], the increasing incidence has been attributed to the obesity epidemic. In Stockholm, the increase co-occurs with a substantial increase in the prevalence of overweight and obesity. The rising incidence could largely be attributed to a rise in BMI; our estimations suggest the incidence of diabetes would have remained stable 1990–2010, if the prevalence of overweight had not increased. This is a crude estimation, based on the assumption that the relative excess mortality in people with diabetes does not differ by BMI level. On the other hand, obesity is known to increase the risk of type 2 diabetes seven-fold [23] and it is difficult to imagine a scenario where such a dramatic increase in the prevalence of obesity as seen in Stockholm, is not reflected in diabetes incidence. Physical inactivity also became more frequent which may have contributed the BMI development, although the influence of inactivity per se on the incidence trends was limited. On the positive note, prevalence of smoking in Stockholm decreased substantially over the last two decades. However, since smoking is a much less potent risk factor for diabetes than overweight [24], this decline would not been enough to counterbalance the effect of rising BMI. Importantly, the rise in diabetes incidence seemed to curb from 2002 and onwards. One reason may be that the rise in prevalence of overweight was slowing down at the same point in time. Self-reported weight, potentially afflicted with underreporting was used to calculate BMI. Consequently, we may have underestimated the prevalence of overweight/obesity and its influence on the diabetes trends.

In contrast to our findings, previous Swedish studies indicate stable incidence of diabetes 1996–2003 [10], 1972–2001 [5] and even a tendency for a decrease 1990–2007 [9]. Regional differences may account for some of this variation. It is well known that for example the incidence of myocardial infarction has a strong geographic patterning [25]. Trends in risk factors may differ and there may also be differences in access to health care and diagnostic intensity. The Swedish Board of Health and Welfare does not recommend screening for diabetes expect in patients with a high risk of type 2 diabetes, but clinical practice may vary across the country. With regard to Stockholm County, there have been no large scale screening activities during the last 20 years.

The advantages of this study were the population-based design, large sample and long observation period. The major limitation was the use of self-reported diabetes. Still, data from other population- based studies suggest that the validity of such information is high [26]. We found that 76% of subjects reporting diabetes in the 2010 survey could be found in the national drug prescription registry. This fits with data which indicate that 24% of all adult patients with diabetes are treated by diet only [27]. The survey information did not allow us distinguish between different forms of diabetes. Sweden has the second highest incidence of type 1 diabetes in the world and it has been increasing during the last 30 years [28]. On the other hand, type 2 diabetes accounts for 85–90% of all diabetes in Sweden [29] and hence, our findings pertain primarily to this diabetes type. We could not detect undiagnosed diabetes and consequently, diabetes occurrence is underestimated. This will only amplify the trend if the proportion with undiagnosed diabetes has decreased over time, e.g. due to a greater awareness of diabetes over the last 20 years. The improvement in case ascertainment must be substantial in order to explain our results, i.e. if we assume that 1/3 of all cases of diabetes were undiagnosed in 2010, the proportion must have been at least 80% higher in 1990, to account for the rise. New diagnostic criteria, including a lowering of the cut-off levels to define diabetes was adapted in Sweden in 1999. Notably, in women the greatest rise in incidence seems to appear between 1998 and 2002 but in men there was no indication of a sudden rise at that time. Notably, the number of participants of the surveys conducted prior to 2002 was relatively small and the prevalence estimates from these years are consequently subject to more random variability and should be interpreted with caution. With regard to generalizability, it has been shown that participants in the Stockholm Health Surveys tend to have higher income and education and more often be of Swedish origin, than the general population. E.g. in the 2010 survey, 17% of male and 18% of female participants were born outside of Sweden, compared to 21% of all men and 22% of all women living in Stockholm (Statistics Sweden). This leads to underestimation of the occurrence of diabetes. Still, as long as the determinants behind non-response are constant over time, the influence of this bias is minimal. Notably, the increasing prevalence that we observed was supported by data from the prescription registry for the period 2006–2010, and this registry is likely complete with regard to pharmacologically treated diabetes. So is the Stockholm data representative for Sweden as a whole? Comparing prescription data from the Stockholm region to the entire Swedish population indicated that the prevalence of diabetes is higher outside of Stockholm and also that the increase in prevalence was even more pronounced.

## Conclusion

Despite improvements in other areas of public health such as a reduction in the incidence of myocardial infarction [25] and stroke [30], our findings suggest that diabetes has been increasing in Stockholm over the last decades both in terms of prevalence and incidence. The driving force behind this development seems primarily to be increasing levels of overweight and obesity. These findings stress the importance of preventing obesity, in order to reduce the public health burden of diabetes.

## Supporting Information

File S1 Formulas for calculation of incidence and cumulative diabetes risk.
(DOCX)

Table S1 Participants from the Stockholm Public Health Surveys 1990–2010.
(DOCX)
