# Supplementary material for: Prevalence and Incidence of Diabetes in Stockholm County 1990-2010
Source: PLoS One. 2014 Aug 14;9(8):e104033. doi: 10.1371/journal.pone.0104033 (PMC4133405; doi:10.1371/journal.pone.0104033)
Supplement: File S1 — Formulas for calculation of incidence and cumulative diabetes risk. (DOCX) [file pone.0104033.s001.docx]

### Appendix A. Formulas for calculation of incidence and cumulative diabetes risk.

Equation 1. Calculation of age and stratum-specific incidence rate


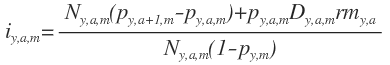


Equation 2. Calculation of standardized incidence rate (standardized against the population in 2010)


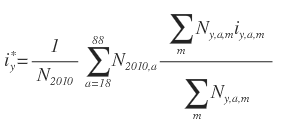


Equation 3. Calculation of cumulative risk of diabetes 18-88 years.


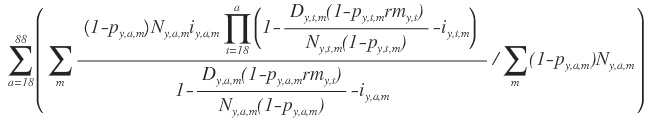


*a*=Age

*D*=No of deaths during the year

*M*=Municipality

*N*=population size at the beginning of the year

*P*=Prevalence of diabetes

*rm*=Relative mortality

*y*=Year
