# Supplementary material for: Prevalence and Incidence of Diabetes in Stockholm County 1990-2010
Source: PLoS One. 2014 Aug 14;9(8):e104033. doi: 10.1371/journal.pone.0104033 (PMC4133405; doi:10.1371/journal.pone.0104033)
Supplement: Table S1 — Participants from the Stockholm Public Health Surveys 1990–2010. (DOCX) [file pone.0104033.s002.docx]

###### Table S1. Participants from the Stockholm Public Health Surveys 1990-2010

| Survey year | No. Men | No. Women | Total |
| --- | --- | --- | --- |
|  |  |  |  |
| 1990 | 1 302 | 1 462 | 2 764 |
| 1994 | 4 190 | 4 817 | 9 007 |
| 1998 | 1 334 | 1 601 | 2 935 |
| 2002 | 13 603 | 16 499 | 30 102 |
| 2006 | 15 457 | 18 888 | 34 345 |
| 2007^a^ | 10 276 | 13 222 | 23 496 |
| 2010^b^ | 32 506 | 41 433 | 73 939 |

^a^ follow-up of participants in the 2002 survey

^b^ Includes participants in the survey of 2010 (new sample) and follow-up of participants in the 2006 and 2007 surveys
